# Establishment of canine nasal carcinoma organoids for drug sensitivity testing and functional characterization of S100A2

**DOI:** 10.3389/fvets.2026.1912335

**Published:** 2026-10-23

**Authors:** Haru Yamamoto, Mohamed Elbadawy, Amira Abugomaa, Ryouichi Tsunedomi, Masahiro Kaneda, Tatsuya Usui, Kazuaki Sasaki

**Affiliations:** 1 Laboratory of Veterinary Pharmacology, Department of Veterinary Medicine, Faculty of Agriculture, Tokyo University of Agriculture and Technology, Tokyo, Japan; 2 AIRDEC Mini Co., Ltd., Tokyo, Japan; 3 Department of Pharmacology, Faculty of Veterinary Medicine, Benha University, Moshtohor, Toukh, Egypt; 4 Faculty of Veterinary Medicine, Mansoura University, Mansoura, Egypt; 5 Department of Epidemiology, Faculty of Veterinary Medicine, Okayama University of Science, Imabari, Japan; 6 Laboratory of Veterinary Anatomy, Department of Veterinary Medicine, Faculty of Agriculture, Tokyo University of Agriculture and Technology, Tokyo, Japan

**Keywords:** anti-cancer drug, canine nasal carcinoma, organoid, S100A, transcriptomic analysis

## Abstract

Canine nasal carcinoma (NC) is highly malignant and progressive, with metastasis. Radiation therapy is a common treatment; however, it has various drawbacks, including cost and anesthetic risks in elderly patients. Therefore, the development of alternative anti-cancer drug therapies has been required. In this study, we established an NC organoid culture method as an experimental model and explored more effective therapeutic targets. NC organoids were generated from patient-derived tissues from diseased dogs with NC. Electron microscopy of NC organoids revealed goblet cell differentiation with mucus granules and luminal structures. Immunohistochemical staining was positive for CK19, E-cadherin, and Ki67, and their expression patterns corresponded to those in the original tissues. Furthermore, NC organoids were tumorigenic in immunodeficient mice. We identified useful supplements for culturing NC organoids and found that each NC organoid line exhibited different sensitivities to anti-cancer drugs. RNA sequencing was performed using samples from healthy dogs and NC organoids. Pathways related to MYC targets, oxidative phosphorylation, and protein secretion were enriched in NC organoids compared with normal nasal cells. Among the differentially expressed genes, S100A2 was selected for further functional investigation based on its increased expression and previously reported involvement in cancer-related processes. S100A2 knockdown reduced cell proliferation and invasiveness, supporting a functional contribution of S100A2 to malignant phenotypes in canine NC. These findings establish canine NC organoids as an experimental platform for investigating canine NC biology and provide a basis for future comparative studies.

## 1 Introduction

Canine nasal carcinoma (NC) accounts for approximately 1–2% of malignant tumors in dogs and is highly malignant, with a malignancy rate of approximately 75% (1, 2). Affected dogs commonly present with upper respiratory signs, such as epistaxis, purulent nasal discharge, and sneezing, often accompanied by destruction of the nasal turbinate or septum. Exposure to environmental chemicals, including cigarette smoke, has been suggested as a risk factor (3). 46% of dogs with NC show metastasis to the lungs or regional lymph nodes (1). The treatment mainly involves surgical resection combined with radiation therapy or chemotherapy (4–7). Radiation therapy is often considered the standard treatment, with a reported median survival time of 8–23 months (4, 8). However, treatment-related toxicities and limited therapeutic efficacy remain the major clinical challenges (9). Prognosis depends on the clinical stage and histopathological subtype; nasal adenocarcinoma is the most common subtype, while squamous cell carcinoma is associated with a poorer prognosis (10, 11).

Organoids have recently been used as a culture method in basic cancer research. This allows the generation of organ-like structures, known as organoids, by culturing epithelial cells derived from organs in Matrigel, a medium that enhances stemness. In humans, culture methods have been established for various organs, including the small intestine and colon (12–14). Organoids can reproduce several structural and biological characteristics of their tissues of origin. Compared with 2D cultures, they offer a more realistic representation of tumor characteristics *in vivo* despite their cost and technical challenges (15). We previously established organoids from patient-derived tissues of canine bladder cancer (16–18), lung cancer (19), malignant mesothelioma (20), and apocrine adenocarcinoma (21). However, no culture method has been reported for canine NC. Similar to dogs, NC is a head and neck tumor in humans (22). In rare cases, the tumor is locally invasive and aggressive. Radiation therapy and chemotherapy have been used in combination with existing surgical treatments, but their efficacy is so limited that it is necessary to develop more effective therapeutic targets. Although cell lines have been established in humans (23), the development of more efficient *in vitro* culture models to elucidate pathogenesis is urgently needed.

In this study, we established patient-derived canine NC organoids and characterized their histopathological features and tumorigenicity. We further evaluated inter-individual differences in anti-cancer drug sensitivity and performed transcriptomic comparisons between NC organoids and normal nasal cells to identify candidate genes associated with malignant phenotypes. Among these candidates, S100A2 was selected for further functional investigation. Given the rarity of human nasal cavity tumors and the limited availability of experimental models, canine NC organoids may also provide a basis for future comparative studies of canine and human NC.

## 2 Materials and methods

### 2.1 Antibodies, case information, and primers

Information regarding the antibodies and cases is listed in Tables 1, 2, respectively. Polymerase chain reaction (PCR) primers were purchased from FASMAC Co., Ltd. (Kanagawa, Japan; Table 3).

**Table 1:** List of primary and secondary antibodies.

Application	Primary antibody	company	Catalog no.	Ratio
IHC	CK19	Abcam	NB100-687SS	1/100
	E-cadhelin	BD bio sciences	610181	1/100
	Vimentin	Santa cruz	sc-373717	1/200
	Ki67	NOVUS	NB600-12520	1/100
	MUC5AC	Bioss	bs7166R	1/100
	S100A2	Genetex	GTX121457	1/100
Application	Primary antibody	company	Catalog no.	Ratio
Western blotting	VCP	GeneTex	GTX113030	1/1000
	S100A2	GeneTex	GTX121457	1/500
Secondary antibody	Company	Catalog no.	Ratio	
Anti-Rabbit secondary antibody	Bio-techne	042–206	-	

**Table 2:** NT case info.

Patient no.	Age	Sex	Breed
NT21001	12y 3m	MC	Miniature Schnauzer
NT21002	9y	MC	Miniature Schnauzer
NT21004	12y 2m	MC	No info.
NT21005	7y 5m	FS	Borzoi
NT21006	13y 6m	M	Shiba
NT22010	11y 8m	F	Airedale Terrier
NT23006			
Tannpopo			

**Table 3:** Primerd for real-time quantitive PCR analysis.

Target	Forward	Reverse
Dog S100A2	GATGGGAGACCTGGATGAGA	GGGGAGTCCTGGAAGAAGTC
Dog c-MYC	AAAAAGCCACCGCGTACATC	TCCGCAACAAGTCCTTTTCG
Dog GAPDH	AACTCCCTCAAGATTGTCAGCAA	CATGGATGACTTTGGCTAGAGGA

### 2.2 Organoid culture and passaging

NC organoids were prepared from dogs with NC, and NC tissues were isolated during surgery. All samples were collected from animal clinics in Japan and rapidly transferred to our laboratory in preservation buffer (Table 4). This study was approved by the Institutional Animal Care and Use Committee of the Tokyo University of Agriculture and Technology (approval number: 0020007). Tissue processing to generate organoids and passaging was performed as previously described (16, 24). The extracted tissue was washed 3 times with 3 mL of Phosphate-Buffered saline (PBS) and minced finely with scalpels and scissors on ice. Minced tissues were then enzymatically digested in 15-mL tubes containing 0.125 mg/mL of both collagenase type II and dispase II solution (Liberase TH, Sigma Aldrich, America) diluted by Advances Dulbecco’s Modified Eagle Medium (DMEM) (Thermo Fisher Scientific, Tokyo, Japan) for 30 min (with pipetting every 15 min) at 37 °C using a thermostatic shaking water bath (25). The tubes were centrifuged at 200 g for 5 min; the supernatants were discarded, and the pellets were trypsinized for 5–10 min at 37 °C using TrypLE solution (Thermo Fisher Scientific). The obtained cell suspensions were passed through 100 μm nylon cell strainers (Falcon, Cary, NC, USA) to remove undigested tissues and fibers, neutralized with fetal bovine serum (FBS), and centrifuged at 600 g for 3 min. The supernatants were discarded, and the cell pellets were washed thrice with 8 mL of PBS. Appropriately sized cell pellets were separated, mixed with Matrigel (BD Biosciences, America), and added to 24-well plates (40 μL/well). The plates were incubated at 37 °C for 30 min in a CO_2_ incubator. After Matrigel polymerization, 500 μL of culture medium was added to each well. The organoids were maintained at 37 °C in a 5% CO_2_ incubator, and the culture medium was renewed every other day. The NC organoids were passaged every 1–2 weeks at a 1:3–4 ratio. For passaging the NC organoids, 5 mM Ethylenediaminetetraacetic acid (EDTA) (Thermo Fisher Scientific)/PBS was added to each well, the plates were kept on ice for 90 min, and the gel domes were gently disrupted at 30 min. Organoid suspensions were collected, transferred to 15-mL tubes, and centrifuged at 600 g for 3 min. The supernatants were aspirated and discarded, and the organoid pellets were washed with cold PBS and dissociated into single cells using pre-warmed (37 °C) TrypLE solution for 5 min, mixed with Matrigel, and seeded as described above. The composition of the culture medium of the NC organoids is presented in Table 4. All animal experiments were performed in accordance with the relevant guidelines and regulations.

**Table 4:** Supplement.

2x supplement					
Name	Company	Catalog number	Stock concentration	Amount	Final concentration
Advanced DMEM/F12 (1x)	Gibco	12634–010		500 mL	
HEPES	Dojindo	342–01375	1M	1 mL	2 mM
GlutaMAX-1 (100x)	Gibco	35050–061		10 mL	
Nicotinamide (NIC)	Sigma	N0630	1M	10 ml	20 mM
NAC	LKT Laboratories	A0918	500 mM	2 mL	2 mM
A83-01	Adooq Bioscience	A12358	5 mM	10 ul	1 uM
Penicillin–Streptomycin Solution (100x)	WAKO	168–23191		10 ml	
NT media					
Name	Company	Catalog number	Stock concentration	Amount	Final concentration
L-WNR conditioned medium as a source of 50% Wnt, Noggin and R-Spondin	Prepared in Lab from L-WRN cells (CRL-3276TM)			25 mL	
2x supplement				25 ml	
EGF	PeproTech	AF-100-15	500 ug/ml	5 ul	50 ng/mL
Primocin	Nacalai tesque	14860	50 mg/mL	100 ul	100 ug/ml
Preservation buffer					
Name	Company	Catalog number	Stock concentration	Amount	Final concentration
Advanced DMEM/F12 (1x)	Gibco	12634–010		500 ml	
HEPES	Dojindo	342–01375	1M	1 ml	10 mM
GlutaMAX-1 (100x)	Gibco	35050–061		5 ml	
Penicillin–Streptomycin Solution (100x)	WAKO	168–23191		5 ml	

### 2.3 RNA sequencing

Based on the QC results, the libraries were appropriately pooled and loaded onto the next-generation sequencing platform, NovaSeq 6000 (Illumina). Sequencing was carried out according to the manufacturer’s instructions with a 150 bp paired-end configuration, yielding approximately 78 GB per pool. Reads were mapped to the reference genome (canFam3: https://www.ncbi.nlm.nih.gov/datasets/genome/GCF_000002285.3/) using the STAR (version 2.5.1b) software (26). Unstranded fragment counts and transcripts per million (TPM) were obtained using RSEM (version 1.3.3) (27), followed by the removal of genes with low counts and normalization with the trimmed mean of M-values method (28) using the TCC package (version 1.38.0) (29, 30) for the R software (version 4.2.2; R Foundation for Statistical Computing, Vienna, Austria). The edgeR (version 3.40.2) [19910308, 22287627] package was used to identify the differentially expressed genes (DEGs) based on a false discovery rate q-value threshold <0.05. Genes with at least a two-fold change and a q-value < 0.05 were considered differentially expressed and visualized using a volcano plot. Using normalized count data, gene set enrichment analysis (GSEA, version 4.3.2 with Molecular Signatures Database v2024.1) was performed as described previously (19).

### 2.4 Hematoxylin and eosin staining

NC organoids and their tumor tissues were histopathologically analyzed by hematoxylin and eosin (H&E) staining as previously described (31). After organoids or tissues were fixed with 4% paraformaldehyde (FUJIFILM Wako Pure Chemical Corporation) for 24 h at room temperature (RT), they were embedded in paraffin and sliced into 4-μm-thick sections using a sliding microtome (REM700; Co., Ltd. Yamato Koki Industry, Japan). After deparaffinization, the slides were stained with Mayer’s hematoxylin solution (FUJIFILM Wako Pure Chemical Corporation) and eosin solution (Muto Pure Chemicals Co., LTD., Tokyo, Japan), following standard procedures. Images were captured using an optical microscope (BX-43; Olympus, Tokyo, Japan) and analyzed blindly by three veterinary pathologists.

### 2.5 Wright–Giemsa staining

The cell suspension was applied to a glass slide to create a smear. The cell smear was fixed with 100% methanol (Fujifilm Wako Pure Chemical Corporation), mounted with mixed Wright–Giemsa stain (Giemsa Stain Solution, Fujifilm Wako Pure Chemical Corporation, Tokyo, Japan; Wright Stain Solution, Muto Pure Chemicals Co., LTD., Tokyo, Japan), and stained for 30 min. Images were obtained using a light microscope (BX-43; Olympus).

### 2.6 Immunohistochemical staining

After deparaffinization, the organoids, tissues, and xenograft-derived slides were subjected to antigen retrieval in 10 mM citrate buffer at 121 °C for 5 min. To quench endogenous peroxidase, slides were immersed in 3% hydrogen peroxide (Fujifilm Wako Pure Chemical Corporation) diluted in dH2O for 30 min. After blocking, they were incubated with the primary antibody diluted with PBS at 4 °C overnight. They were then reacted with Envision+Dual Link System HRP for 30 min, and the targeted antigen was detected using 3,3′-diaminobenzidine (Nacalai Tesque, INC., Kyoto, Japan) followed by counterstaining with hematoxylin for microscopic examination. Images were obtained using a light microscope (BX-43; Olympus).

### 2.7 Quantitative real-time PCR

Quantitative real-time PCR was performed as previously described (19, 32). Total RNA was extracted from untreated or drug-treated cells for 72 h, and cDNA was synthesized using the ReverTra Ace qPCR real-time kit (TOYOBO, Osaka, Japan) according to the manufacturer’s instructions. Primer oligos were purified by FASMAC Co., Ltd., and are shown in Table 2. Quantitative real-time PCR was performed using the QuantiTect SYBR I Kit (QIAGEN, Hilden, Germany) and the StepOnePlus real-time PCR System (Applied Biosystems, Carlsbad, CA, USA).

### 2.8 Transmission electron microscope

Transmission electron microscope (TEM) was performed as previously described (20, 33). Briefly, NC organoids were transferred into a 15-mL Falcon tube and fixed with 2.5% glutaraldehyde (adjusted 20% glutaraldehyde (NACALAI TESQUE, INC., Kyoto, Japan) by 0.1 M PBS) at 4 °C overnight. The NC organoids were then washed with 0.1 M cacodylate (pH 7.4) and incubated for 2 h at 4 °C in 0.1 M sodium cacodylate (pH 7.4) solution containing 2% osmium tetroxide and 1.5% potassium ferrocyanide. Thereafter, NC organoids were washed with PBS, dehydrated in a graded ethanol solution, and embedded in Epon. Ultrathin sections (70–110 nm) were obtained using an ultramicrotome (Leica UC7, Wetzlar, Germany) with diamond knives, and loaded onto 50-mesh copper grids coated with a formvar/carbon film. Sections were post-stained with uranyl acetate and lead citrate for 15 min at RT. TEM images were obtained at the Applied Medical Research Laboratory (Osaka, Japan).

### 2.9 Mouse xenograft assay

All animal experiments were conducted with permission from the Animal Ethics Committee of Tokyo University of Agriculture and Technology (approval number: R04-120). Organoid xenograft experiments were performed as previously described (18, 34). Briefly, after tumor cells were seeded in 24-well plates with Matrigel and passaged into new wells after 1–2 weeks of culture, they were used for xenografting. 1 × 10^6^ NC organoid cells (passage 2–3) were mixed with a cold injection medium (organoid medium containing 50% Matrigel, 10 μM Y-27632, and 10 μM CHIR99021) on ice. The cells were subcutaneously implanted into the thigh region of SCID mice (5-week-old, male, 25 g average weight; SANKYO Laboratory, Tokyo, Japan) under inhalation anesthesia with isoflurane (induction at 3–5% and maintenance at 1.5–2.5% in oxygen; Zoetis Japan, Tokyo, Japan). Three weeks later, mice were euthanized by overdose inhalation of isoflurane (>5%) followed by confirmation of death through cessation of respiration and heartbeat, and tumor tissues were collected for histopathological analysis and compared with the original tumor tissues. The xenograft tissue did not metastasize to any other region. A tumor size exceeding 1000 mm^3^ was the criterion for euthanasia, but none of the mice were euthanized. The studies were performed in accordance with the animal research: reporting of *in vivo* experiments guidelines.

### 2.10 Anti-cancer drugs sensitivity test

The sensitivity of NC organoids to anti-cancer drugs was analyzed as previously described (17, 19). Briefly, 2 × 10^3^ NC organoid cells were prepared, mixed with 10 μL of Matrigel, and seeded in 96-well plates. The cells were then treated with different anti-cancer drugs, namely, carboplatin (1–100 μg/mL), doxorubicin (1–100 nM), and toceranib (2 ~ 8 μM) for 72 h. The concentration ranges were selected based on previously reported clinical dosing and pharmacokinetic studies in dogs (35–38) and were designed to cover concentrations sufficient to generate dose–response curves for IC₅₀ determination. Three days after drug treatment, 10 μL of PrestoBlueTM cell viability reagent (Thermo Fisher Scientific) was added to each well and incubated for 4 h in a CO_2_ incubator. Cell viability was evaluated by measuring fluorescence intensity using a microplate reader (TECAN, Männedorf, Switzerland). The data were plotted using SigmaPlot (Systat Software Inc.). The half-maximal inhibitory concentration (IC50) was measured for each drug, and sensitivity was visualized as a heatmap.

### 2.11 S100A2 siRNA transfection

Cells from NC organoids were seeded at a density of 1 × 10^5^ cells in a 6-well plate and cultured for 24 h. Subsequently, they were transfected with small interfering RNA (siRNA) against *S100A2* or negative control siRNA (Table 5) using Lipofectamine RNAiMAX (Invitrogen) dissolved in Opti-MEM (Invitrogen) at a final concentration of 40 nM. After 72 h, the cells were collected, and cell proliferation, cell invasion, and quantitative PCR analyses were conducted.

**Table 5:** SiRNA.

siRNA		Sequence	Company
siRNA-1	s	UGGGAAAUCUGGAUGAGAA	Nippongene
	as	ACCCUUUAGACCUACUCUU	
siRNA-2	s	CUCUAGCUUUCCCUUUUGU	Nippongene
	as	GAGAUCGAAAGGGAAAACA	
siRNA-3	s	AGGCGGAGAUGAAGACACU	Nippongene
	as	UCCGCCUCUACUUCUGUGA	

### 2.12 Cell proliferation assay

NC organoid cells were mixed with Matrigel and seeded in 96-well plates (2000 cells/10 μL gel/100 μL medium/well). The number of living cells on days 1, 4, and 7 was measured and analyzed by quantifying the fluorescence intensity of PrestoBlue cell viability reagent using a microplate reader, as previously described (18, 34).

### 2.13 Cell invasion assay

Membranes were coated with 2% Matrigel to provide an extracellular matrix barrier for assessing invasive capacity, and NC organoid cells were seeded at a density of 3 × 10^4^ cells in the upper chamber, with or without the drug. To induce cell migration, 600 μL of 2.5D organoid medium (18, 34) was added to the lower chamber. All media were removed after 24 h, and the membranes to which the cells migrated or invaded were fixed with methanol for 15 min and stained with Giemsa solution (FUJIFILM Wako Pure Chemical Corporation). The number of cells that invaded the membrane was counted.

### 2.14 Statistical analysis

Data are shown as mean ± S. E. M. Statistical analysis was conducted using SigmaPlot using the two-tailed Student’s t-test, Welch t-test, Pearson correlation analysis, or Mann–Whitney U test. *p* values < 0.05 were considered statistically significant. All confidence intervals were set at 95%.

## 3 Results

### 3.1 Generation of canine NC organoids derived from patient dogs

A schematic of this study is shown in Figure 1A. NC organoids were established using tissue samples obtained from dogs with intranasal tumors. Normal nasal mucosal cells from healthy dogs were collected and subjected to Giemsa staining (Figure 1B). Normal cells exhibited slow proliferation, limiting their ability to sustain long-term culture. In contrast, NC organoids formed spherical structures within 1–2 weeks (Figure 1C). Transmission electron microscopy revealed well-developed microvilli in intercellular spaces and luminal surfaces. Undifferentiated cells, goblet cells with mucus granules, and squamous/intermediate cells with tonofilaments were also observed, indicating structural differentiation similar to that of the native tissue (Figure 1D). Optimization of the culture medium components identified essential supplements for NC organoid maintenance (Supplementary Figure S1).

**Figure 1:**
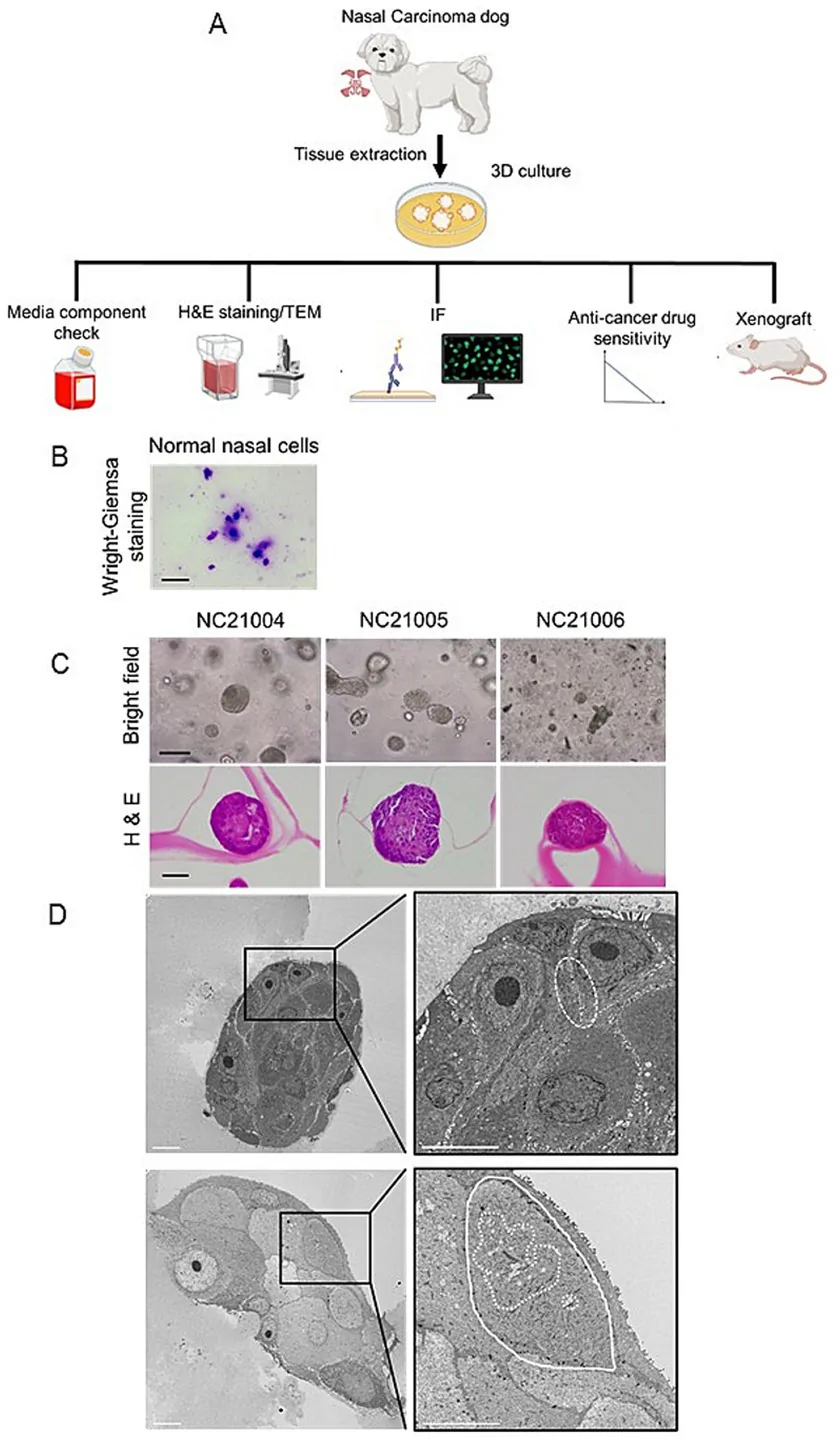
Generation and characterization of canine NC organoids. **(A)** Schematic overview of the experimental workflow for the establishment and analysis of canine nasal carcinoma (NC) organoids. The schematic was created using BioRender. **(B)** Normal nasal epithelial cells collected from the nasal cavity of healthy dogs using cotton swabs and stained with Wright–Giemsa. **(C)** Representative bright-field images (scale bsssswar: 200 μm) and hematoxylin and eosin (H&E) staining (scale bar: 50 μm) of NC organoids derived from three independent cases (NC21004, NC21005, and NC21006). **(D)** Ultrastructural analysis of NC organoids using transmission electron microscopy (TEM). Scale bar: 100 μm.

### 3.2 Tumorigenicity of NC organoids

NC organoids were subcutaneously transplanted into mouse thighs and harvested after three weeks (Figure 2A). Morphological comparison of pre-implantation organoids, original tissues, and xenografts revealed similar histopathological features, including solid structures, variable nuclei, and mitotic activity (Figure 2B). Immunohistochemistry analysis demonstrated the positive expression of cytokeratin 19, E-cadherin, and Ki67, confirming the maintenance of epithelial identity and proliferative capacity in NC organoids (Figure 2C).

**Figure 2:**
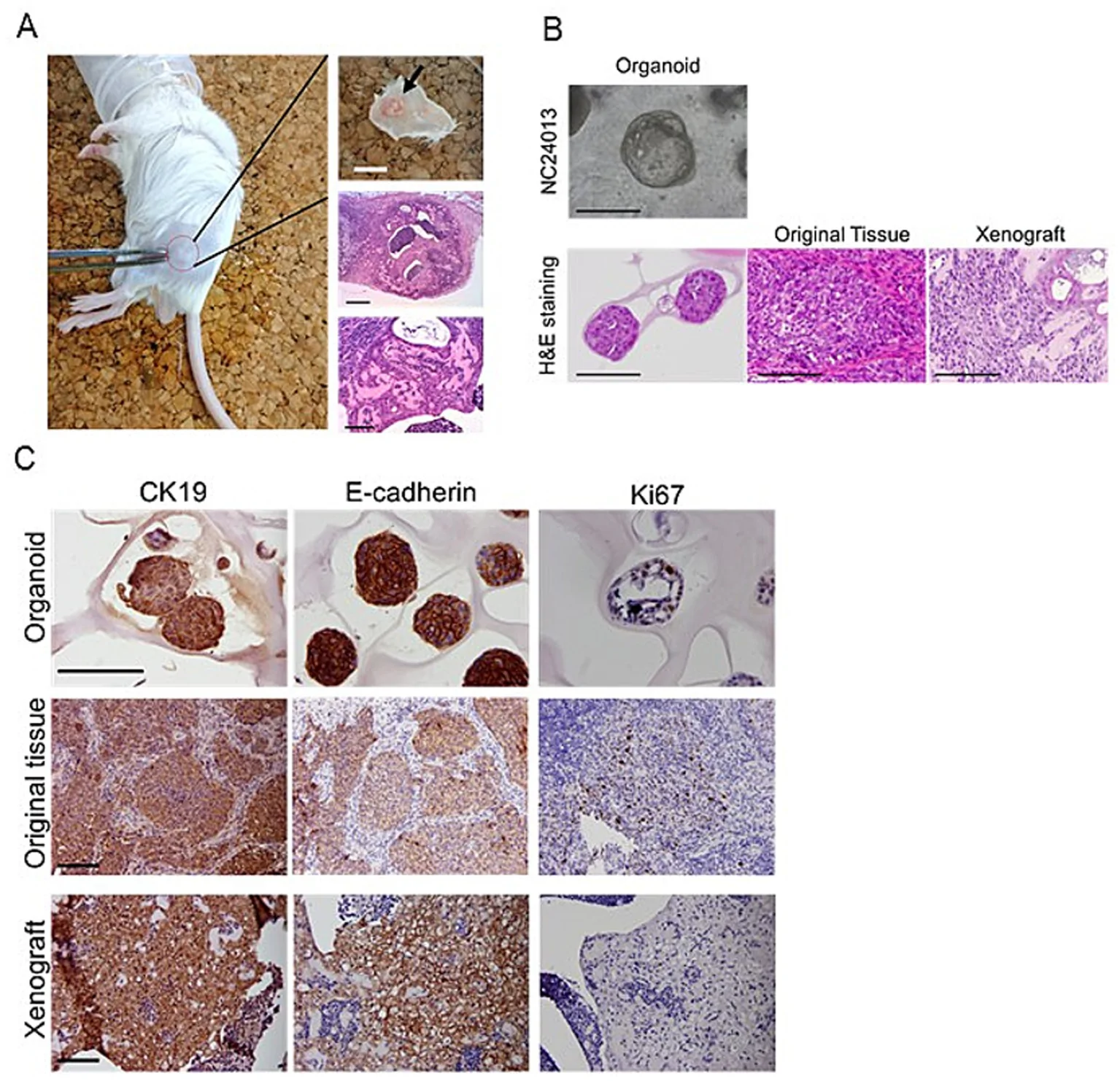
Tumorigenicity of canine NC organoids in mice. **(A)** Nasal carcinoma (NC) organoids were transplanted subcutaneously into immunodeficient mice to evaluate their tumorigenic potential. The xenografted tumor tissue (indicated by a black arrow) was excised and subjected to hematoxylin and eosin (H&E) staining. **(B)** Representative images of NC organoids, their corresponding original tumor tissues, and xenografts (*n* = 4). Scale bar: 100 μm. **(C)** Expression of the adenocarcinoma marker cytokeratin 19 (CK19), the epithelial marker E-cadherin, and the proliferation marker Ki67 was assessed by immunohistochemistry (IHC) and compared among NC organoids, original tumor tissues, and xenografts. Scale bar: 100 μm.

### 3.3 Sensitivity of NC organoids to anti-cancer drugs

To evaluate the therapeutic responses, NC organoids from six cases were treated *in vitro* with doxorubicin, carboplatin, or toceranib. Variable sensitivities were observed among NC organoid lines, demonstrating inter-individual differences in drug responses. These results suggest that NC organoids may provide an experimental platform for investigating heterogeneity in anti-cancer drug sensitivity (Figure 3).

**Figure 3:**
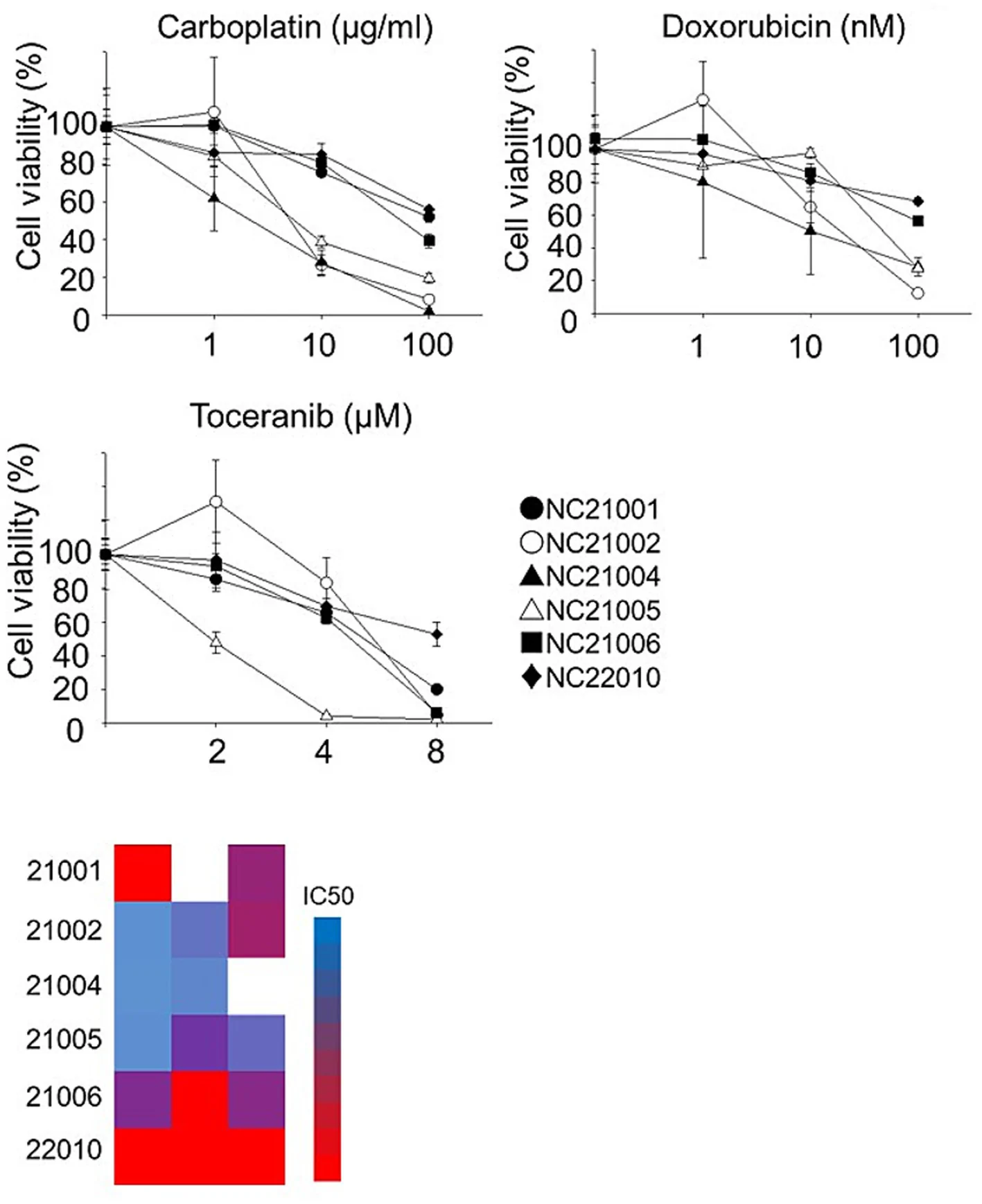
Sensitivity profiles of canine NC organoids to three anti-cancer drugs. Nasal carcinoma (NC) organoids were treated with the commonly used chemotherapeutic agents carboplatin, doxorubicin, and toceranib. Cell viability was assessed using a PrestoBlue cell viability assay. Data are presented as mean ± S.E.M. (*n* = 3). The heatmap shows the IC₅₀ values for each organoid line, with blue indicating high sensitivity and red indicating low sensitivity.

### 3.4 Identification of therapeutic targets in NC organoids via RNA sequencing

RNA sequencing and GSEA were performed using normal nasal cells (*n* = 4) and NC organoids (*n* = 6) (Figure 4A). PCA revealed two distinct clusters that separated the normal and carcinoma-derived cells (Figure 4B). Volcano plots revealed 5,707 differentially expressed genes (normal > NC: 2,521; NC > normal: 3,286) (Figure 4B). GSEA indicated that pathways related to MYC targets, oxidative phosphorylation, and protein secretion were enriched in the NC organoids (Figure 4C). Among the differentially expressed genes, S100A2 showed markedly higher expression in NC organoids than in normal nasal cells. S100A2 was selected for further functional investigation based on its differential expression and previously reported involvement in cancer cell proliferation and invasion. Consistent with the RNA-seq findings, S100A2 expression was also markedly higher in NC tissues than in normal nasal tissues (Figure 4D).

**Figure 4:**
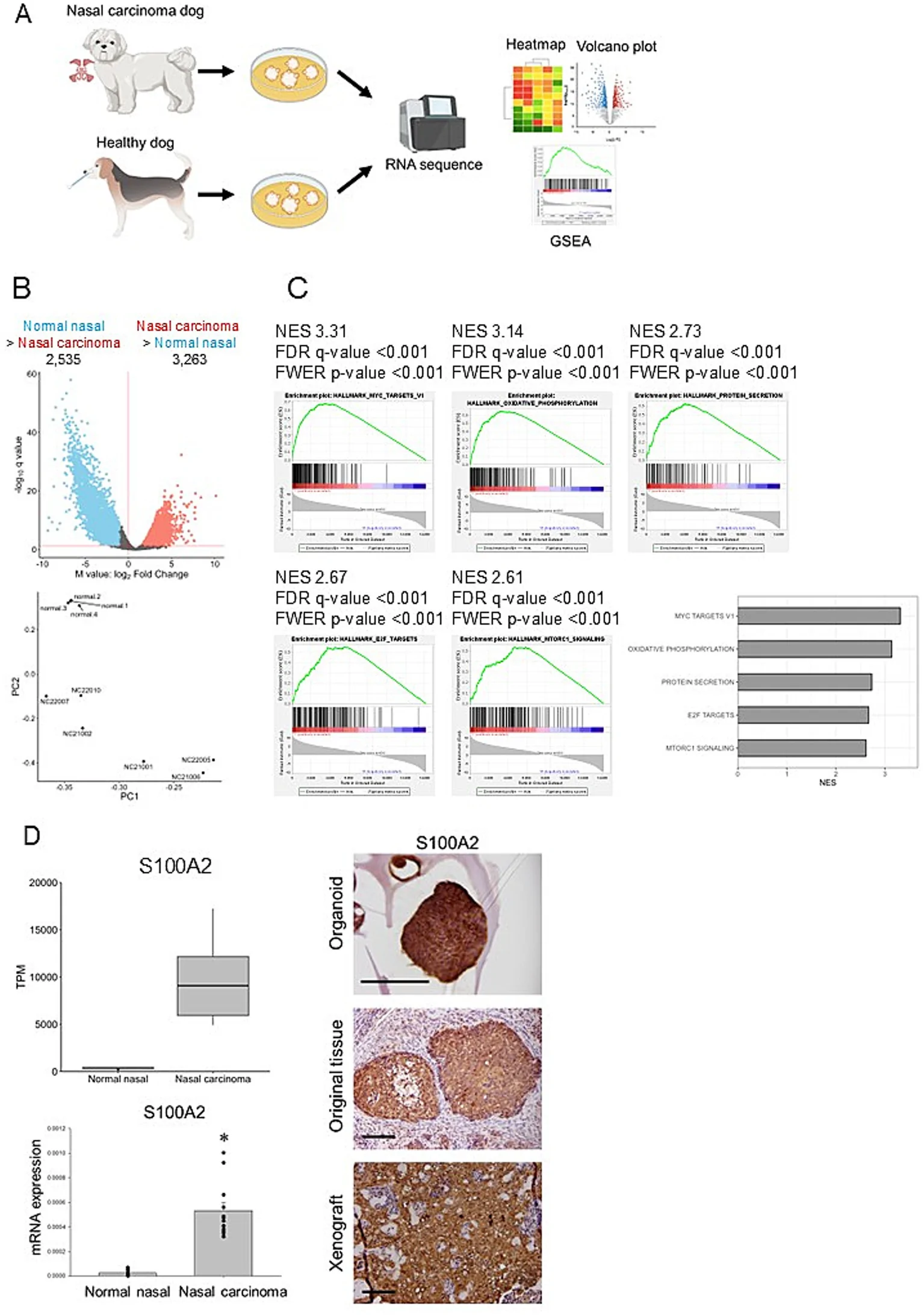
Identification of potential therapeutic and prognostic markers in NC organoids by RNA sequencing. RNA sequencing was performed on nasal carcinoma (NC) organoids (*n* = 6) and normal nasal cells (*n* = 4). Experimental workflow is shown in **(A)** (created with BioRender). **(B)** A volcano plot showing the differential gene expression between NC organoids and normal nasal cells. Cyan points (2,521) represent genes significantly downregulated in NC organoids compared to normal nasal cells [M value < −1, q-value < 0.05, mean of TMM-adjusted count > 50 in normal nasal cells, coefficient of variation (CV) < 1 in each group]. Salmon points (3,286) indicate genes significantly upregulated in NC organoids compared to normal nasal cells (M value > 1, q-value < 0.05, mean of TMM-adjusted count > 50 in NC organoids, CV < 1 in each group). A principal component analysis (PCA) plot demonstrating a clear separation between the two groups. **(C)** Gene set enrichment analysis (GSEA) identified six pathways that were more highly activated in NC organoids than in normal nasal cells. Relative enrichment is shown as a normalized enrichment score (NES) plot. **(D)** Among the upregulated genes, *S100A2*, a member of the S100 family, was further examined by RNA-seq (transcripts per million, TPM) and qPCR. qPCR data are expressed as mean ± S.E.M. NC organoids (*n* = 6) vs. normal nasal cells (*n* = 4). Statistical significance was determined using the Mann–Whitney U test. *p < 0.05 vs. normal nasal cells. *S100A2* expression was also confirmed in NC organoids, the corresponding original tumor tissues, and xenografts by immunohistochemistry (scale bar: 100 μm).

### 3.5 Role of S100A2 in NC organoid behavior

To investigate the function of *S100A2*, NC organoids were transfected with three distinct siRNAs, and the knockdown efficiency was confirmed by western blotting (Figure 5A). *S100A2* knockdown significantly suppressed cell proliferation (Figure 5B) and invasiveness (Figure 5C). However, no statistically significant change in c-MYC mRNA expression was detected following S100A2 knockdown (Figure 5D). Given the limited sample size (*n* = 4), this result should be interpreted cautiously and does not exclude a potential relationship between S100A2 and MYC signaling. A schematic summary of the contribution of S100A2 to NC organoid proliferation and invasiveness is shown in Figure 5E.

**Figure 5:**
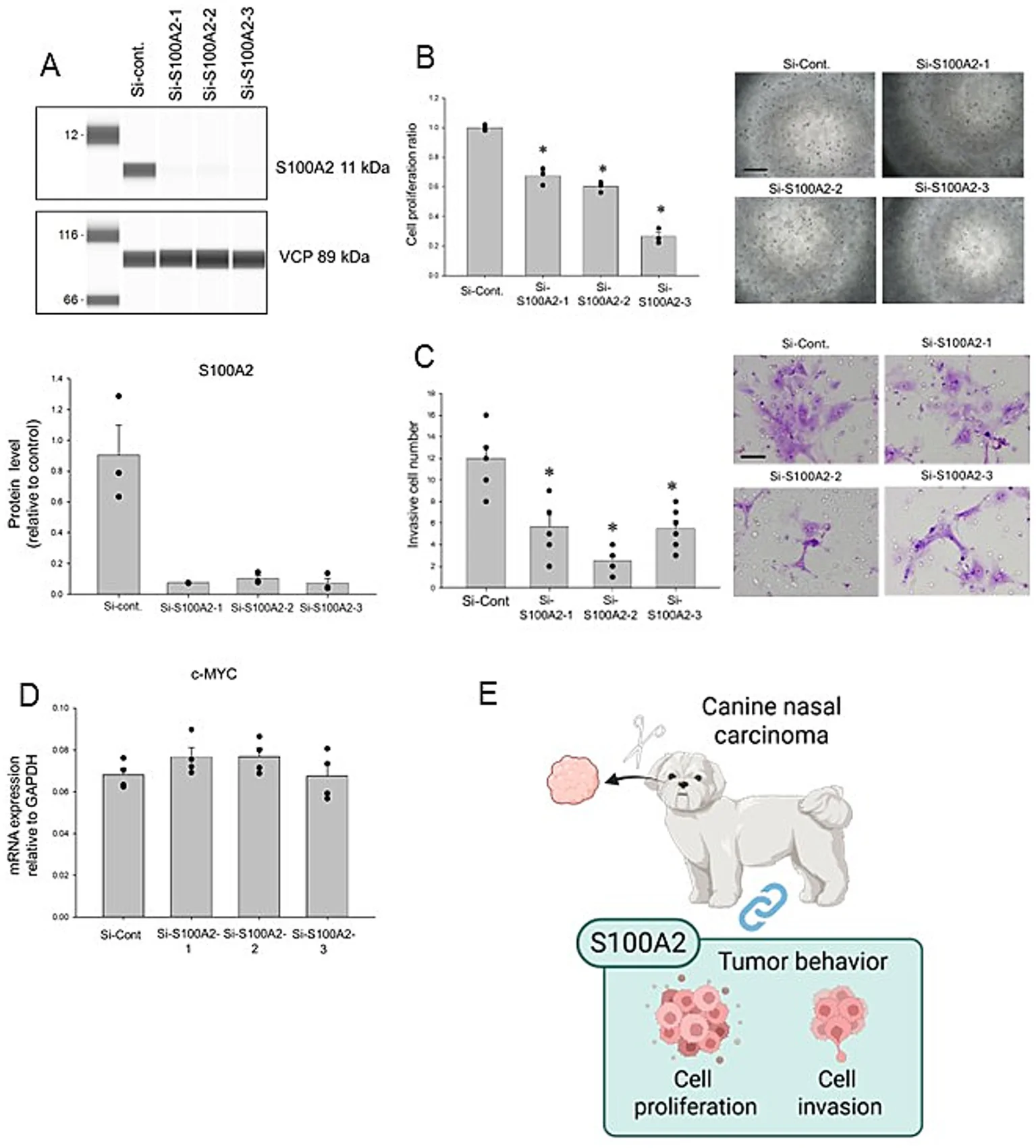
Effects of *S100A2* knockdown on NC organoids. Knockdown efficiency was confirmed by western blotting **(A)**. Data are presented as the mean ± S.E.M. from three independent experiments (*n* = 3). The effect of *S100A2* knockdown on cell proliferation was assessed using a cell proliferation assay. Data are presented as the mean ± S.E.M. from three independent experiments (*n* = 3) using a single patient-derived NC organoid line. *p* < 0.05 vs. Si-Cont, two-tailed Student’s t-test. Scale bar: 500 μm **(B)**. The effects of *S100A2* knockdown on organoid invasiveness were assessed using a cell invasion assay. Data are presented as the mean ± S.E.M. from six independent experiments (*n* = 6) using a single patient-derived NC organoid line. *p* < 0.05 vs. Si-Cont, two-tailed Student’s t-test. Scale bar: 50 μm **(C)**. *cMYC* expression was measured by quantitative polymerase chain reaction (qPCR). Data are presented as the mean ± S.E.M. from four independent experiments (*n* = 4) using a single patient-derived NC organoid line **(D)**. A schematic summary (created using BioRender) illustrating the contribution of *S100A2* to NC organoid malignant behavior, including proliferation and invasiveness **(E)**.

## 4 Discussion

In this study, we established, for the first time, methods for culturing canine NC organoids derived from patients and demonstrated their usefulness as a culture model. Additionally, we demonstrated that canine NC organoids can be used as an experimental platform to investigate inter-individual differences in anti-cancer drug sensitivity. Furthermore, transcriptomic and functional analyses suggest that S100A2 contributes to the malignant behavior of canine NC, including cell proliferation and invasiveness.

An advantage of the organoid platform is its capacity for expansion and longer-term experimental manipulation, allowing repeated pharmacological and molecular analyses using patient-derived tumor cells. However, the present study did not compare organoid drug responses with clinical treatment outcomes or primary-tissue assays. Therefore, its predictive value for therapeutic selection remains to be established.

NC has occurred not only in dogs, but also in humans, and there have not been many cases, accounting for approximately 0.5 ~ 1% of all tumors (39, 40). However, the 5-year survival rate is approximately 30%, and more than three-quarters of cases are malignant, with squamous cell carcinoma being the most common (41). Human NC can be categorized as a head and neck tumor, and its symptoms are similar to those in dogs, including nosebleeds, sneezing, and facial deformities. Due to its high local invasiveness in the region near the skull, complete surgical extraction is difficult. NC treatment involves a combination of radiation therapy (42) and chemotherapy (43, 44). Chemotherapy varies depending on the stage of the tumor, but its effectiveness has been limited (43, 45), and it makes it difficult to achieve remission. Therefore, the search for new therapeutic targets and the development of culture models are needed. Although organoids have been established for head and neck cancers such as tongue and pharyngeal cancers (23), there are no models focused on human NC, and other useful culture models are scarce (46). Considering the similarities between canine and human NC in disease progression and histological characteristics, canine NC organoids may provide a basis for future comparative studies. However, direct cross-species molecular comparisons are required to determine whether the molecular characteristics and therapeutic vulnerabilities are conserved between canine and human NC. Furthermore, although the NC organoids retained key histopathological and epithelial characteristics of the parental tumors, comprehensive molecular fidelity was not evaluated in this study. Future studies should include matched molecular profiling of parental tumors, organoids, and xenografts to further evaluate model fidelity.

RNA sequencing of canine NC organoids and nasal mucosal cells derived from healthy dogs revealed significant differences in the gene expression profiles. GSEA revealed enrichment of pathways related to cell proliferation and growth in canine NC organoids compared with normal nasal cells. However, the normal nasal cells were collected by cotton swab and could not be maintained in long-term culture, whereas the NC samples were analyzed as organoids. Therefore, differences in cellular composition, culture conditions, and proliferative state, in addition to tumor status, may have contributed to the observed transcriptomic differences. Accordingly, the identified DEGs cannot be interpreted exclusively as NC-associated alterations.

Among the genes identified in this comparison, S100A2 showed significantly higher expression in canine NC organoids and was therefore selected for further functional investigation. *S100A2* is one of 20 members of the S100 family and a calcium-binding secreted protein (47). The S100 family has been reported to be related to tumor proliferation, metastasis, and invasion (48–50). Meanwhile, reports have shown that *S100A2* is downregulated as a tumor suppressor gene in some tumors such as oral and liver cancers (51, 52). This suggests that S100A2 functions differently across cancer types and may exert either inhibitory or pro-proliferative effects depending on the tumor context (47). However, its role remains unclear, and this is the first report of its relationship with canine NC. Previous studies have shown that S100A2 promotes tumor proliferation, metabolism, invasion, and metastasis through several mechanisms, including GLUT1 and PI3K/AKT/mTOR-related signaling (31, 48–50). In the present study, S100A2 knockdown significantly reduced the proliferation and invasiveness of NC organoids, supporting a functional role of S100A2 in these malignant phenotypes. However, the downstream signaling mechanisms were not directly investigated in this study. Moreover, *c-MYC* mRNA expression was not significantly altered, although the limited sample size (*n* = 4) may have reduced statistical power. Further studies examining signaling changes following S100A2 perturbation are required to clarify the underlying molecular mechanisms. Because the functional effects of S100A2 knockdown were evaluated in a single patient-derived NC organoid line, further validation across multiple independent organoid lines is required to determine the generalizability of these findings.

In conclusion, we developed a canine NC organoid culture method and investigated the relationship between this disease and *S100A2* expression. Thus, we suggest that *S100A2* plays a role in NC cell proliferation and invasion. In the future, canine NC organoids can be used in comparative studies to evaluate conserved mechanisms not only in dogs but also in humans.

## Data Availability

The datasets presented in this study can be found in online repositories. The names of the repository/repositories and accession number(s) can be found in the article/Supplementary material.
