# Supplementary material for: Establishment of canine nasal carcinoma organoids for drug sensitivity testing and functional characterization of S100A2
Source: Front Vet Sci. 2026 Oct 23;13:1912335. doi: 10.3389/fvets.2026.1912335 (PMC13741214; doi:10.3389/fvets.2026.1912335)
Supplement: Supplementary file 1 [file Image_1.pdf]

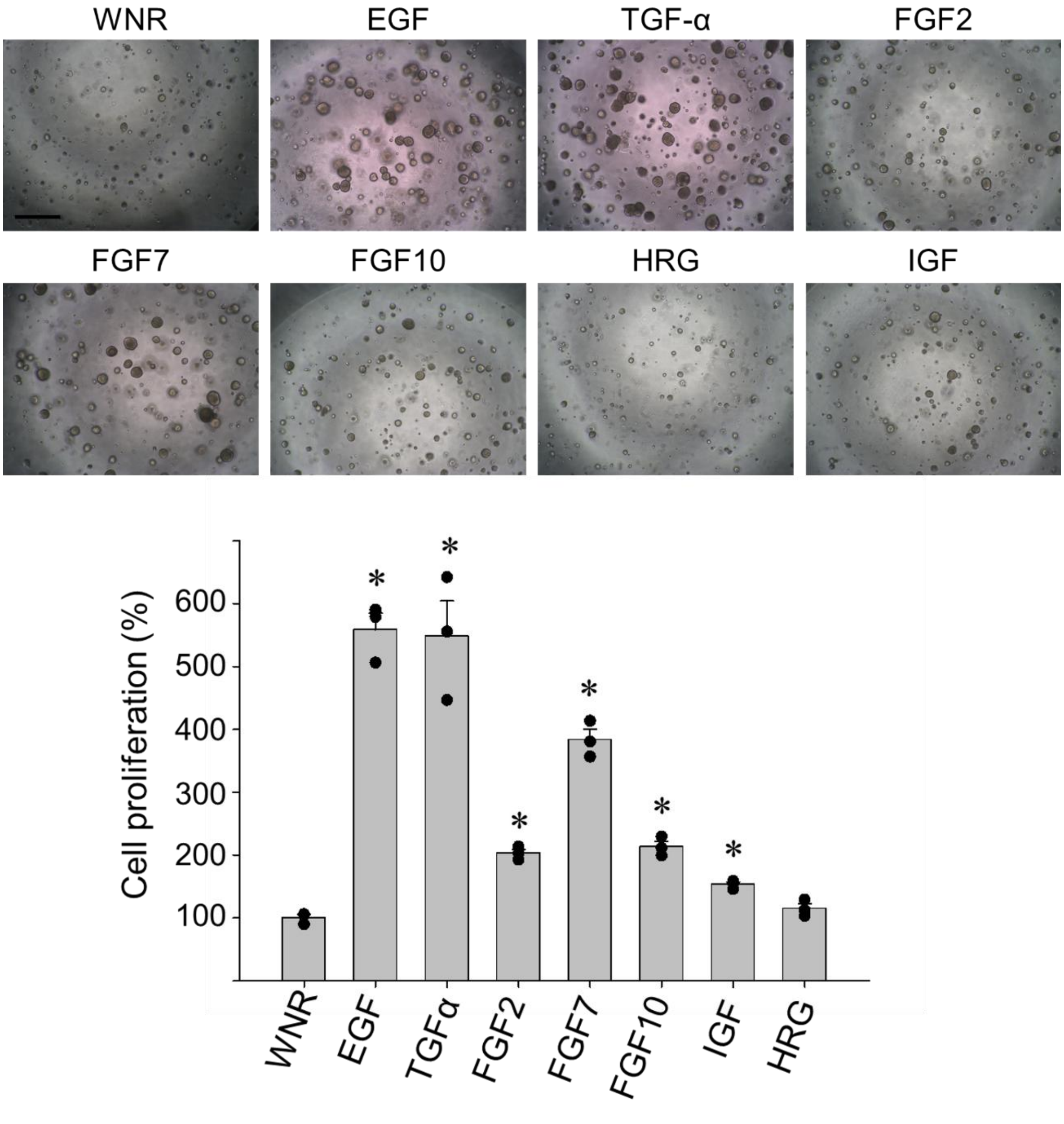

Fig. S1. Screening of effective medium supplements for canine nasal carcinoma (NC) organoids. (A) Representative bright-field images of NC organoids cultured with various growth supplements. Scale bar: 500 µm. (B) Cell proliferation of NC organoids

treated with each supplement was evaluated using a cell proliferation assay. Data are presented as mean ± S.E.M. ($n = 3$). $*p < 0.05$ vs. control.
